# Simultaneous Spectrophotometric Determination of Trigonelline, Diosgenin and Nicotinic Acid in Dosage Forms Prepared from Fenugreek Seed Extract

**DOI:** 10.22037/ijpr.2019.1100790

**Published:** 2020

**Authors:** Neda Mohamadi, Mostafa Pournamdari, Fariba Sharififar, Mehdi Ansari

**Affiliations:** a *Pharmaceutics Research Center, Institute of Neuropharmacology, Kerman University of Medical Sciences, Kerman, Iran. *; b *Department of Medicinal Chemistry, Faculty of Pharmacy, Kerman University of Medical Sciences, Kerman, Iran. *; c *Herbal and Traditional Medicines Research Center, Department of Pharmacognosy, Kerman University of Medical Sciences, Kerman, Iran. *; d *Departments of Pharmaceutics, Faculty of Pharmacy, Kerman University of Medical Sciences, Kerman, Iran.*

**Keywords:** Fenugreek, Simultaneous determination, Spectrophotometry, Herbal Dosage Forms, Trigonelline, Diosgenin, Nicotinic Acid

## Abstract

Mathematical algorithms offer a useful method for quantitative analysis of compounds in multi-component mixtures to overcome the overlapping problems occurred in UV spectrophotometry. The aim of this study is to develop a method for simultaneous determination of bioactive compounds in herbal dosage forms produced from fenugreek extract. A UV- spectrophotometric method based on mathematical algorithm was used to simultaneous determination of trigonelline (TRG), diosgenin (DI), and nicotinic acid (NA). The maximum absorbance (λ_max_) was determined to be 232.65 nm, 296.23 nm, and 262.60 nm for TRG, DI, and NA, respectively. The calibration curves showed good linearity for all analytes in the concentration range of 1–20 μg/mL (R^2^=0.9995, 0.9997, 0.9994 for TRG, DI and NA, respectively). The Intra- and inter-day precisions were in the range of 1.1-10.7% and 1.2-8.2%, respectively. The accuracy of the method was 96.0% for TRG, 92.9% for DI, and 104.2% for NA. The limits of detection (LOD) and quantification (LOQ) were found to be 0.91 and 3.06 µg/mL for TRG, 0.99, and 3.30 µg/mL for DI and 0.33 and 1.10 µg/mL for NA. The validated method was applied for determination of the analytes in the tablet, capsule and thin film dosage forms prepared from the fenugreek seed extract. The mean recovery percentages of the analytes were in the range of 90.0-97.4%, 85.6-105.4%, and 90.0-99.0% for tablet, capsule, and film dosage forms, respectively. Generally, the validated method could be a good candidate for routine spectrophotometric determination of the analytes without any necessity for pre-analysis extraction.

## Introduction

Bioactive compounds in medicinal plants can be considered as initial candidates for new drug discovery (1). Phytotherapy is a definite part of primary health care, so ingredient assay in herbal product seems to be necessary (2). The herbal ingredients have been used in the traditional and modern medicines, nutraceuticals, and dietary supplements. Efficacy and safety of herbal products directly depend on the chemistry of medicinal herb. Phytochemical stability of herbal medicines is provital to their effectiveness (3). 

*Trigonella foenum-graecum *L., commonly known as fenugreek (Shanbalileh in Persian), is an annual plant belongs to Fabaceae family. There is a long history for the usage of the leaves and seeds of fenugreek in Iranian traditional medicine as general tonic and anti-diabetic remedy (4). The seeds of the plant have hypoglycemic, anti-oxidant, anti-inflammatory (5, 6), and anti-nociceptive effects (7, 8). Fenugreek dermal patch has been effective for management of inguinal hernia post-operative pain (9). Polyphenol compounds (e.g., isovitexin and rhaponticin), alkaloids (e.g., trigonelline; TRG), and sapogenin (e.g., diosgenin; DI) seem to be the major bioactive ingredients in fenugreek seeds (10). In this study, TRG and DI have been selected for simultaneous determination due to known pharmacological effects and significant amount in the plant seeds.

**Figure 1:**
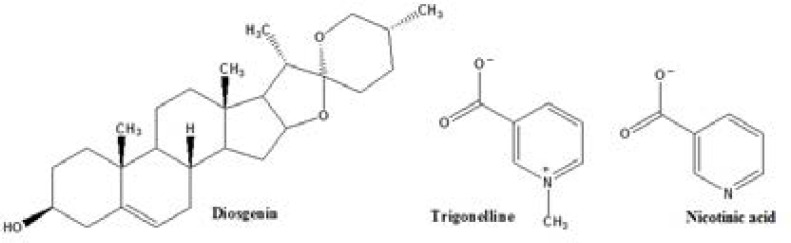
Chemical structures of trigonelline, diosgenin and nicotinic acid

TRG is a pyridine alkaloid (Figure.1) and a vitamin B3 (nicotinic acid; NA) derivative (11) which possess many therapeutic properties such as neuroprotective, anti-diabetic, hypolipidemic, anti-cancer, and anti-mutagenic activity (12). DI is a steroid sapogenin present in various plants e.g. *Costus speciosus*, *Smilax menispermoidea*, *Aletris*, *Trigonella*, and many species of *Dioscorea* (13). It has estrogenic activity (14) and also is a precursor of the progesterone synthesis so that it has been previously administered as contraceptive pills (15).

Herbal pharmacologic biomarkers were frequently used for quality control of medicinal plants.

Development of validated and reliable analytical methods for identity and purity of herbal ingredient has not been progressed with the rapidly increasing number of ingredients in the market. Lack of validated analytical methodologies in tune with complex ingredient chemistry suggests the much needed research in this area. Among the various experimental techniques, chromatographic methods are commonly used for quantitative and quantitative analysis which is a part of medicinal herbs quality control. In general, these methods are difficult to optimize and they are time-consuming and costly; therefore, the present study aims to develop a simple, sensitive, accurate, time-saving, and inexpensive UV-spectrophotometric method for simultaneous determination of TRG, DI, and NA in various herbal dosage forms.

## Experimental

*Chemical and Instruments*

Standard TRG and DI were purchased from Sigma Company (USA); and standard NA was prepared from Merck Company (Germany). All used solvents were of high purity and analytical grade. A Lambda ^TM^ 25 UV–Vis spectrophotometer (PerkinElmer, Germany) was used to measure the absorbance of standard and sample solutions.

*Calibration curves*

A primary standard solution with a concentration of 100 µgmL^-1^ of each analyte was prepared; TRG and NA were dissolved in deionized water, while DI was dissolved in 2 mL methanol and then was made to the volume with deionized water. For determination of maximum wavelengths (λ_max_) of standards, the solutions were separately scanned in the range of 200 - 400 nm against blank. Different aliquots were then taken from the stock solution and diluted with deionized water to prepare a serial concentration in the range of 1–20 µgmL^-1^. The calibration curves were constructed by plotting absorbance *vs* concentration.

**Method validation**

*Accuracy and precision*

A mixed aqueous solution of analytes (10 µgmL^-1^ of each analyte) was scanned in the range of 200-400 nm using UV spectrophotometer. The absorbance of each individual compound and a mixture of three analytes were measured at three determined λ_max_ (232.65 nm, 296.23 nm and 262.60 nm); then, the concentration of all analytes were calculated by using the following equations.

Where *A*_mi_ is the absorbance of the mixture at three wavelength of λ_1 (232.65),_λ_2(296.23),_λ_3(262.60)_.

Intra- and inter-day precision and accuracy were investigated by analysis of six mixed solution of analytes in concentrations of 1, 2, 4, 8, 10, and 20 µg/mL (three independent replicates). Precision levels were expressed as the relative standard deviation (RSD%) (n = 18).

*Recovery Studies*

Recovery percentage of the method was calculated based on the following formula with measuring the concentration of three analytes in spiked and non-spiked samples: 

Recovery= (X_s_-X_ns_)/X_ad_ ×100

Where X_s_ is the mean result of spiked samples, X_ns_ is the mean result of non-spiked samples and X_ad_ is the amount of added analyte (10µg/mL). The recovery percentage of each analyte for dosage forms was carried out in three replicates (totally n = 9) and the results were reported as Mean ± SD.

*Limit of detection (LOD) and limit of quantitation (LOQ)*

LOD and LOQ were estimated as 3.3×SD_b_ ⁄ Slope and 10× SD_b_ ⁄ Slope respectively, where SD_b_ is the standard deviation of the blank samples (n = 25).

*Preparation of dosage forms*

The validated method was applied for simultaneous determination of three analytes in three herbal dosage forms including the tablet, capsule and bucoadhesive thin film containing *Trigonella foenum-graecum *seed extract.  The dosage forms were prepared based on the thesis of Pharm.D students of Kerman University of Medical Sciences (16-18).

*Estimation of the analytes in the real samples*

Ten tablets and capsules content were separately weighed, powdered, and extracted with 80% ethanol by sonication method for 30 min. The solutions were filtered through whatman filter paper, and the filtrates were diluted to 100 μg/mL and the samples were analyzed using proposed method. Ten thin films were extracted with warm water by sonication method for 30 min. The extract was centrifuged at 5000 rpm for 15 min. The supernatant was diluted to prepare a 100 μg/mL concentration and analyzed by proposed method.

## Result and Discussion

Many analytical techniques including UV-spectrophotometry (19, 20), GC-MS (21, 22), HPLC (23-25), and HPTLC (26, 27) have been developed and reported for individual determination of TRG, DI, and NA; but simultaneous analysis of these phytochemicals is of high importance for evaluating the quality of herbs and herbal products. 

**Figure 2.**
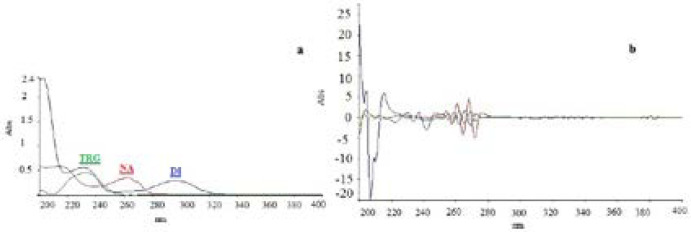
Zero-order (a) and Third-derivative (b) UV spectra of standard TRG, DI and NA at concentration of 10 µg/mL

UV-spectrophotometry as a simple analytical method usually requires specific low-overlapped spectra of chemicals for simultaneous analysis of multi-component mixture (28). Spectrophotometry based on mathematical algorithm helps to overcome the spectral overlapping problems (29). In the current work, the λ_max_ of TRG, DI, and NA was determined to be 232.65 nm, 296.23 nm, and 262.60 nm, respectively (Figure.2a). TRG and NA showed no absorbance at 296.23 nm. Hence, this wavelength is selective for DI determination by zero-order spectrophotometry. However, there were no other wavelengths at which TRG and NA can be determined selectively due to high level of overlapping among the spectra. The same problem was found for the second derivative spectra of these compounds. A third derivative spectrum was provided for simultaneous determination of TRG at presence of DI at 253.8. But due to overlapping of NA spectrum with the spectra of the other two, a mathematical algorithm system of three variables was used to calculate the analytes concentration in a multi-component mixture (30) (Figure. 2b).

*Calibration curve*

The method was linear in the concentration range of 1-20 µg/mL of the analytes with an acceptable regression coefficient (R^2^ equivalent to 0.9995, 0.9997, and 0.9994 for TRG, DI, and NA respectively). The values of standard deviation for slope and intercept are very low and near to zero which indicates high precision of the method. The results are summarized in Table 1.

*Accuracy and precision*

The mean recoveries and CVs are illustrated in Tables 1 and 2. The results showed an acceptable accuracy and precision over the entire examined concentration range. The validity of the proposed method was more assessed by recovery study *via* standard addition test as explained in the experimental section. The mean recovery percentages of the analytes were in the range of 90.0-97.4%, 85.6-105.4%, and 90.0-99.0% for tablet, capsule, and film dosage forms respectively. Intra-day precision (as repeatability) and inter-day (as intermediate precision) were in the range of 2.3-10.7%, 1.1-7.9%, and 4.6-8.2% for TRG, DI, and NA respectively. The results have been shown in Table 2.

*LOD and LOQ*

LOD and LOQ of the analytes were determined as 0.91and 3.06 µg/mL for TRG, 0.99, and 3.30 µg/mL for DI, and 0.33 and 1.10 µg/mL for NA (Table 1). 

*Determination of the analytes in the herbal dosage forms*

The results of determination of the analytes in herbal tablet, capsule, and thin film are shown in Table 3. The concentration of TRG, DI, and NA in different formulations respectively ranged 6.00-7.31 µg/mL, 1.60-3.60, and 2.00-6.90 µg/mL. 

**Figure 3.**
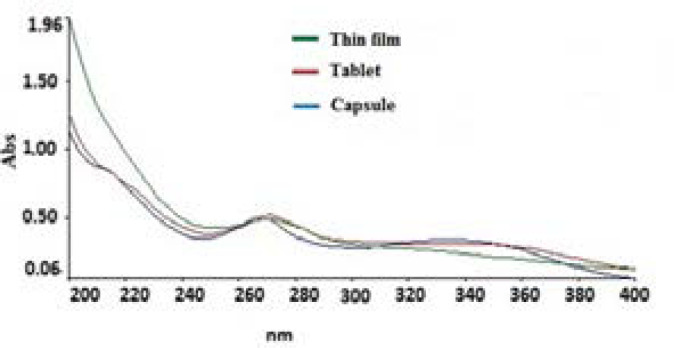
UV-absorption spectra of 100 µg/mL of the table, capsule and thin film dosage forms of fenugreek extract

The log P of DI is about 6.34 and is very lipophilic compound and the low amount of DI in the thin film dosage form must be due to its low solubility in aqueous medium utilized in exaction process to prepare the thin film sample (Figure 3). 

Laila *et al*. in 2014 suggested an HPTLC method for the simultaneous determination of quercetin and DI in fenugreek extract (31). A sensitive and reproducible TLC method has been reported by Trivedi *et al*. 2007 for quantitative analysis of DI. They reported DI value in the range of 0.53% (w/w) in the fenugreek seed powders, 0.087% (w/w) in the fenugreek leaf powder, 0.015 and 1.27% (w/w) in the fenugreek stem powder and extract, respectively, and 0.586% (w/w) in a formulation containing fenugreek seed powder (32). In current study, DI was determined in the range of 1.6% (w/w) in the thin film, an aqueous extract of fenugreek seeds, and 3.3-3.6 % (w/w) in the tablet and capsule which contain containing ethanolic extract of plant seeds. Chopra *et al*., 2007 applied a spectrophotometric and HPTLC method for the determination of TRG in pharmaceutical formulations (vaginal tablets and bioadhesive vaginal gels). As this report indicated, the sample recoveries from all formulations were accorded with their respective label claims (19). A rapid liquid chromatography–mass spectrometry method has been reported for the simultaneous quantification of TRG and NA in coffee by Perrone *et al*., 2008 (33). Content of TRG was found 1.03% (w/w) in the green coffee samples.

**Table 1:** Linearity, accuracy, LOD and LOQ parameters of the spectrophotometry method

**Parameters**	**TRG**	**DI**	**NA**
Slope ± SD	0.0430±0.0001	0.0285± 0.0003	0.0045±0.0003
Confidence limits of slope (95%)	0.0427;0.0426	0.0285;0.0285	0.0045;0.0044
Intercept± SD	0.020±0.0002	0.0003± 0.0002	-0.0127±0.0001
Confidence limits of intercept (95%)	0.0201;0.0199	-0.0002;-0.0004	-0.0143;-0.0146
Correlation coefficient (R^2^)	0.9995	0.9997	0.9994
Accuracy%	96.0	92.9	104.2
LOD (µg/mL)	0.91	0.99	0.33
LOQ (µg/mL)	3.06	3.30	1.10

**Table 2:** Precision of the spectrophotometry method at two intra- and inter-day levels for each analyte (1- 20 μg/mL) at the presence of the other two analytes (4 μg/mL).

**Conc. (µg/mL)**	**Intra-day (RSD%)**			**Inter-day (RSD%)**		
	**TRG**	**DI**	**NA**	**TRG**	**DI**	**NA**
1	10.7	3.6	5.8	7.5	7.9	5.5
2	7.7	6.1	7.9	6.9	6.8	8.0
4	2.6	2.5	6.7	1.2	2.4	4.6
8	2.9	1.1	8.2	3.1	2.8	8.2
10	2.6	1.2	6.8	3.8	1.8	6.7
20	2.3	2.4	6.6	2.3	2.5	5.3

**Table 3:** Determination of TRG, DI, NA in herbal dosage forms (100 µg/mL) (n=9)

**Dosage Forms**	**TRG**		**DI**		**NA**	
	**Mean ± SD (µg/mL)**	**Recovery (%)**	**Mean ± SD (µg/mL)**	**Recovery (%)**	**Mean ± SD (µg/mL)**	**Recovery (%)**
Tablet	6.10±1.10	97.4±2.6	3.60±0.39	101.5±2.1	2.30±1.46	90.0±1.1
Capsule	7.31±1.37	101.2±3.3	3.30±0.09	85.6±3.9	6.90±0.25	105.4±2.5
Thin film	6.00±1.42	99.0±1.7	1.60±0.25	90.0±2.3	2.00±1.48	91.7±1.0

## Conclusion

Overall, a precise, accurate, and linear UV spectrophotometry method based on mathematical algorithms was developed for simultaneous determination of trigonelline, diosgenin, and nicotinic acid in herbal dosage forms prepared from fenugreek seed extract. The method was simple as well as low-cost which could be a good candidate for routine UV spectrophotometry determination of these analytes without any necessity for intensive pre-analysis extraction of the samples. 
